# A phenomenological study of the lived experiences of partner relationship breakup during pregnancy: Psychosocial effects, coping mechanisms, and the healthcare providers' role

**DOI:** 10.3389/fgwh.2023.1048366

**Published:** 2023-04-17

**Authors:** Abel Negussie, Eshetu Girma, Mirgissa Kaba

**Affiliations:** ^1^ Department of Social and Population Health, Yirgalem Hospital Medical College, Yirgalem, Ethiopia; ^2^ School of Public Health, College of Health Sciences, Addis Ababa University, Addis Ababa, Ethiopia

**Keywords:** pregnant women, relationship breakup, psychosocial, stress, coping, support, phenomenology, Ethiopia

## Abstract

**Background:**

Pregnancy is a state of continuous changes in feelings and emotions, and highly stressful experiences such as a relationship breakup during this period may result in additional stress for the woman, making pregnancy and motherhood challenging. This study aimed to investigate pregnant women's lived experiences of partner relationship breakup during pregnancy, their coping mechanisms, and the role of healthcare providers in breakup cases during their Antenatal care visits.

**Methods:**

A phenomenological study approach was followed to seek an understanding of the lived experiences of pregnant women who encountered partner relationship breakup. The study was carried out in Hawassa, Ethiopia, and eight pregnant women were involved in in-depth interviews. The data meanings found from participants' experiences were described in a meaningful text and organized into themes. Key themes were developed in reference to the research objectives, and thematic analysis was used to analyze the data.

**Results:**

Pregnant women in such situations faced serious psychological and emotional distress, feelings of shame/embarrassment, prejudice and discrimination, and severe economic struggles. To cope with this multifaceted situation, pregnant women sought social support from family/relatives or close friends, and if they had no other options, from supporting organizations. The participants also revealed that they received no counseling from healthcare providers during their Antenatal care visits, and there was no further discussion to address their psychosocial problems.

**Conclusions:**

Community-level information, education, and communication should be initiated to aware communities about the psychosocial consequences of relationship breakup during pregnancy, address cultural norms and discrimination, and promote supportive environments. Women's empowerment activities and psychosocial support services should also be strengthened. In addition, the need for more comprehensive Antenatal care to address such unique risk conditions is indicated.

## Introduction

Pregnancy is a unique biological event in a woman's life (1), and it is a time in which the woman may feel a great need for protection and emotional support (2, 3). It is well-recognized that pregnancy is a state of continuous changes in feelings and emotions caused by the interaction of physiological, hormonal and emotional factors (4–6). Indeed, highly stressful experiences such as a relationship breakup during this period, especially during the first pregnancy, can result in additional stress for the woman, potentially triggering anxiety and depressive symptoms or disorders in pregnant women.

Context plays a crucial role in gender norms, and sociocultural and economic disadvantages. Patriarchal norms and expectations about how a “good girl or woman” should behave and sociocultural beliefs may also have great implications for women's physical and mental health (7). Similar to other traditional societies, in Ethiopia, gender norms and deep-rooted cultural beliefs, attitudes, and practices that support and promote stereotypes and prejudices against women are common. Ethiopian women also suffer from the impacts of many traditional practices and gender-based violence (8). In addition, despite some progress towards gender equality, especially in urban areas, women in Ethiopia have low levels of empowerment in the economic, political, social, and psychological domains (9). As a result, Ethiopian women often face different and more basic economic constraints than men and the economic dependence of women on men is one of the major causes of social problems. Studies also indicated women in Ethiopia still lag men on several important economic resources and opportunities, including employment rate, earnings, and wage income (10).

Cultural norms and gender roles shape women's perceptions, beliefs, and expectations about pregnancy (11, 12). In many African cultures, particularly in Ethiopia, pregnancy is highly valued to be shared with a spouse or husband, and a pregnant woman who has experienced a partner breakup may be concerned about failing to meet this societal value, which, when combined with other socioeconomic adversities, can lead to psychological or emotional stress. Furthermore, since pregnancy before marriage is considered shameful and unacceptable, single pregnant women may face a revolting stigma. Thus, breaking up during pregnancy has a profound influence on a woman's life in most cultural contexts of low socioeconomic settings, such as Ethiopia, and results in a woman being alone during a time that society generally indicates should be shared with a spouse. This imposes situational stress, which aggravates an already anxiety-provoking situation.

Depression and anxiety during pregnancy have been linked to adverse maternal and fetal health outcomes (13–15). And partner support may be considered doubly important since it affects the mothers' health and wellbeing as well as the birth outcomes of their unborn children (6, 16–19). A breakup with the partner, instead, asserts stress to a woman, making pregnancy and motherhood challenging. The ability to handle with this situation successfully is dependent on coping mechanisms, family relationships, and preconceived notions about both pregnancy and relationship breakup. In this respect, social support is an important protective factor in a woman's ability to cope with stressful events and recover from such events, and thus some types of stress are probably to be tolerable for women who have good social support (20–23).

Antenatal care (ANC) provides the opportunity to interact with and support pregnant women at a critical time in the course of a woman's life. Ethiopia developed a national ANC guideline by adopting the 2016 WHO model of eight contacts to reduce maternal and perinatal mortality and morbidities (24). The guideline focuses on key guiding ANC principles, such as pregnant woman-centered care, maternal health assessment during initial and subsequent contacts, and holistic care approaches to address each woman's social, emotional, physical, psychological needs and expectations and make pregnancy a healthy and positive experience for a woman (24). It is important to pay attention to women who appear to lack protection from a partner, and identifying such pregnant women at risk would allow us to follow them up during their pregnancy and implement necessary therapeutic and psychosocial interventions as needed. If healthcare providers (HCPs) are aware of the significance of some specific situations for psychosocial and emotional burdens in pregnancy, they may be able to more easily identify them and assist with their condition (25, 26).

Although some studies have examined into the effect of partner relationship satisfaction during pregnancy (2, 27, 28), none have assessed into the psychosocial effects of partner relationship breakup during pregnancy. Understanding this specific context helps to make program and policy recommendations for pregnant women who require special care and psychosocial support. To that end, this study focuses on the lived experiences of partner relationship breakup during pregnancy, pregnant women's coping mechanisms, and the role of HCPs in such situations.

## Methods

### Study design and setting

A phenomenological study approach was used to seek an understanding of the lived experiences of pregnant women who encountered partner relationship breakup. The study was conducted in Hawassa city, Sidama National Regional State, Ethiopia, from March 1–30, 2021. Hawassa is 275 kilometers from Addis Ababa and is divided into 8 sub-cities and 32 *kebeles* (lowest administrative units) (21 urban and 11 rural). The estimated total population of the city is 402,903, and the annual estimated number of pregnancies is 13,940. There are a number of local, national, and international non-governmental organizations (NGOs) in the city that provide social and child support services (29).

### Study participants

Different potential institutions, including the Women and Children Affairs office (at the city and sub-city levels), NGOs involved in providing social support services, and health facilities (Antenatal care clinics), were contacted to trace the eligible pregnant women. As a result, eight participants were identified: two from health institutions, four from NGO living houses or shelters (Mother Teresa's Home, Kale-Hiwot Church Child Development Project), and two from Haikdar and Mehal-Ketema sub-cities Women and Children Affairs offices. The potential eligible pregnant women were then approached and given detailed information about the study, and all agreed to participate in in-depth interviews.

All of the pregnant women were separated from their partners during their pregnancy and were married or living as a cohabiting couple prior to the breakup. Pregnant women with a history of gender-based violence or under the age of 18 years were not eligible for the study due to the different additional contexts of these situations, which were not the focus of this specific study.

### Data collection procedure

In-depth interviews (IDIs) were conducted to explore the lived experiences of pregnant women who had encountered a partner relationship breakup. A topic guide developed through a review of relevant literature was used during the IDIs (Supplementary File S1). The participants were asked and probed about their experiences and feelings regarding partner breakup, living conditions, how they tried to manage the situation, and their experiences during health facility contacts for ANC service.

Data collection and analysis were undertaken simultaneously. The IDIs were conducted in Amharic and lasted approximately 45 min on average. In order to achieve data saturation, repeated visits and interviews with participants were conducted, emerging concepts were included, and major themes were compared across interviews. The IDI audio recordings were transcribed verbatim in order to retain the “contexts”, with sound files encrypted and stored in a password-protected folder. All audio-taped interviews were fully transcribed to Amharic and then translated into English for analysis.

### Data analysis

A line-by-line initial open coding of the data was done using Opencode software version 4.02, and the list and descriptions of the developed codes were summarized in a codebook (Supplementary Table S2). The coded data were analyzed by describing the range of participants' experiences.

A thematic analysis based on a descriptive phenomenological approach (30) was employed to identify data patterns with specific meanings and potential implications. To explore and develop themes, data meanings were identified and organized into patterns by reading and rereading raw data. The data meanings identified from participant experiences were described in a meaningful text organized in themes, and discussed in relation to research objectives through applying reflective writing.

### Trustworthiness of the study

To ensure the trustworthiness and quality of the study, the four-dimension criteria (credibility, transferability, dependability, and confirmability) (31) were considered. Through strong engagement and creating trust with the research participants, a rich description of the phenomenon was made. Context-specific descriptions were made based on participants' current living place (personal home, living with family, or living in a shelter) to increase the transferability of the study. Unique descriptions were also considered in the analysis with adaptive approaches.

Verbatim transcription was used to allow for detailed analysis of contexts, and feedback on the transcribed data was acquired from some of the participants to validate the interpretations of their responses. All the data was collected and transcribed by the principal investigator. The transcription reports were read by all authors in order to identify themes in relation to the research objectives, and discussions were held to select final themes and relevant illustrative quotes.

### Ethical considerations

Ethical clearance was obtained from the ethical review committee of the School of Public Health, Addis Ababa University. A letter of support from Addis Ababa University, School of Public Health, was communicated to the appropriate institutions and government bodies. Written informed consent was secured from all participants. Participation in the study was entirely voluntary, and the purpose of the study was clearly explained to study participants prior to data collection.

The interviews took place in settings that guaranteed maximum privacy. Privacy and confidentiality of the information were also ensured both during and after data collection. Three pregnant women who showed severe emotional or psychological distress and didn't receive any counseling or advise were referred to professional counselling. All pregnant women were also advised of regular ANC follow-up.

## Results

### Description of the study participants' background profile

In this study, eight pregnant women from breakup cases were involved in in-depth interviews. All of the pregnancies were unintended, and two of the women had one previous child from their separated partner; for the other six women, it was their first pregnancy. Five study participants were living together as couples with their spouses before the breakup, and the other three were married women. The most common reason for breakup among married women was disagreement over personal issues, while denial or refusal to accept fatherhood was the most common reason among cohabiting couples. Moreover, four of the participants were living in shelters at the time of the interviews (Table 1).

**Table 1:** Background profile of the study participants, March 2021, Hawassa, Ethiopia, (*n* = 8).

Characteristics	Participants							
	P1	P2	P3	P4	P5	P6	P7	P8
Age (Years)	21	28	20	24	21	25	19	20
Highest Education								
High School (HS)	HS			HS			HS	
Elementary School (ES)		ES	ES		ES	ES		ES
Spousal status before the breakup								
Married (M)		M		M	M			
Cohabiting Couple (CC)	CC		CC			CC	CC	CC
Parity	0	1	0	0	1	0	0	0
Relationship duration	1.6 years	9 years	1 year	3 years	6 months	1.3 years	10 months	8 months
Current place of living								
Personal Home (PH)		PH		PH				
With family (WF)	WF						WF	
Shelter (SH)			SH		SH	SH		SH

The study findings are presented with detail descriptions based on the identified descriptive themes: indications and actions of psychological and emotional distress conditions, feelings of shame/guilt and embarrassment, prejudice and discrimination, socioeconomic problems, coping mechanisms, and the role of healthcare providers during ANC service.

### Theme 1: indications and actions of psychological and emotional distress conditions

The pregnant women reported that they experienced extreme stress when their relationship with their partner terminated. The participants indicated that this was the most difficult time because of the unexpected dissolution of the partner relationship and the confusion over accepting the pregnancy condition.

*…… I was stressed and didn't tell anyone, not even my friends, about my situation…… I was confused about what to do…… It was difficult for me to accept my pregnancy…… (Age—25; currently living in a shelter)*

Different expressions and actions from the interviews were appreciated in order to understand the presence and level of stress. Three of the study participants mentioned that when they recognized they were pregnant, they attempted suicide and were unable to convince themselves of the situation. Pertaining the breakup, all participants reported feelings of loneliness and disregard.

*……Sometimes I wish my pregnancy time to be very short. It is difficult to be stressed all the time these days. I wish these dark times to pass quickly…… (Age—19; currently living with family)*

Even though most of the participants had lately confirmed their pregnancy, almost all of them stated that they were thinking of abortion. And they declined their decision for different reasons, including high costs, fear of health risks, and the disallowance of the service during the late pregnancy stage.

### Theme 2: feelings of shame and embarrassment

Another common indication among most the interviewees is a loss of confidence in interacting with others and a fear of discussing the condition with family or close friends. This fear of sharing feelings stemmed from the faced stress and the fear of possible negative attitudes in which people may not understand their real situation, as well as worries about being accused of some unfaithful character related to the split–up with one's partner or due to the denial of the child by the partner.

Moreover, four participants highlighted that even if they felt sick related to their pregnancy, they kept their situation secret and tried to seek medical care on their own. This fear of disclosing one's pregnancy status to families and close friends may exacerbate the distressful condition by hampering the chance of getting help or counseling from families and/or close friends. Likewise, another similar action reported by those study participants who were living together as couples with their spouse before the breakup was an effort to spend their pregnancy time very privately in order to keep the pregnancy secret.

*……After a few weeks, my pregnancy became visible, and I abruptly left my family's home without telling them. Then I hid in a guesthouse/pension for more than two weeks. Nobody recognized me, and eventually I began searching for organizations that can help me and came to this shelter. (Age—20; currently living in a shelter)*

Based on the feelings and actions of the participants, it is worth noting that the perceived cultural taboo of being a single pregnant woman was the primary cause of the intense stress condition, which creates anxiety. When a pregnant woman believes it is unacceptable to be a single pregnant woman in the community and she has failed to satisfy that spousal status, she may be imposed in a stressful situation. Furthermore, the participants indicated in their statements that the accidental breakup of the partner relationship is a stressor factor relating the incident to the fate of one's future life.

### Theme 3: prejudice and discrimination

The study participants emphasized that they had experienced various forms of prejudice and discrimination. According to their reports, these prejudices and discriminations were described as uncaring or unsupportive communications, blaming the pregnancy status, and employers refusing to allow for work. These discriminations emanate from deeeply held cultural values at the family, institutional, and community levels, and some participants reported severe types of discrimination.

*………My families don't try to understand my condition, which makes my situation worse. My father usually insults me with disturbing words, especially when he is drunk. I have no other option but to live with their hurtful words and feelings. (Age—21; currently living with family)*

The participants also detailed how prejudices and discriminations, combined with their perceived cultural conviction, restrains them from sharing feelings and concerns, which leads to self-discrimination due to feelings of shame, exacerbating their distress condition. Another issue raised by pregnant women was the prejudice associated with getting and maintaining a job while pregnant. Comparatively, pregnant women who had “married” spousal status and those living in their own home faced less discrimination.

### Theme 4: socioeconomic problems

#### Income and job uncertainty

All the study participants emphasized that the breakup with their spouse during their pregnancy put them at risk of complex economic challenges. Seven of the eight pregnant women were involved in some daily low-income generating activities; and after the breakup, they were forced to live alone due to the absence of the expected mutual sharing of life tasks. However, due to their pregnancy condition, their full-fledged efforts to work and cover living expenses were limited, as it was difficult to maintain an active working status.

*………I have been struggling to work in some daily income-generating activities, such as washing clothes and preparing food for customers. But now, as I am about to give birth, it is difficult for me to continue with my previous active work. Though, I have no means to support my life. (Age—28; currently living in a personal home)*

When asked about their living conditions, two of the pregnant women mentioned that it was difficult to get and maintain a suitable job; and four of them were forced to join shelters due to being unable to get job, particularly at the matured stage of their pregnancy, and being in a desperate living situation.

*………During the early stages of my pregnancy, I had no problem working and earning good money. But, as time passed, finding a suitable job to do became increasingly difficult, and people were also unwilling to allow me for a job. (Age—21; currently living in a shelter)*

#### Concerns about future parenting roles

All participants expressed future parenting concerns, especially about raising a child being a single parent. They also mentioned the future uncertainties of finding a suitable job. Participants, particularly those living in shelters, were unsure and lacked confidence in having future life plans for how to deal with the difficult situation and fulfill future childcare responsibilities.

*………I can stay here for three months after my delivery. But I'm not sure what happens after I leave this shelter, or whether I'll be able to find a suitable job to meet my child's needs. (Age—20; currently living in a shelter)*

Some participants also raised about the psychological effects of being raised by a single parent on their child's personality development. They described this as one issue that makes their parenting role difficult due to the additional requirement of playing a two-parent role.

### Theme 5: coping mechanisms

Although the participants were psychologically and emotionally distressed as a result of the unexpected dissolution of partner relationship and faced discrimination and financial problems, some significant efforts to deal with the condition were reported. However, besides their late health facility visit for ANC service, most of their coping actions were directed toward economic challenges, and no actions were appreciated to get any help or counseling for the extreme stress condition.

In terms of economic encounters, the pregnant womens' intense struggle to live independently demonstrates the difficult coping situation. Some participants preferred to spend their pregnancy time with their families, while others sought support from close friends. Aside from that, some others were forced to look for or apply for aid organizations when it was difficult to find work to pay for rent and other living expenses. Three of the four participants who were living in shelters reported that they had found themselves in a vagrant situation and that living in shelters was a good choice for them.

*……I was working and living with my employers. And when they told me to stop working and look for another job, I was worried because I didn't know anyone else who could help me at the time, and I thought it would be impossible to find another job. Fortunately, while walking alone on the streets, I met a woman who took me to her home. I stayed with her for about two weeks before she finally brought me to this organization. (Age—25; currently living in a shelter)*

Participants who were in shelters at the time of the interview reported receiving assistance and counseling. They also stated that they freely discuss their concerns with other pregnant women in similar situations, and that their level of stress has reduced.

### Theme 6: the role of healthcare providers during ANC visits

Efforts were made during the course of this study to trace the target pregnant women of breakup cases from ANC follow-up records, but both the identification and recording-keeping of single pregnant women were poor. While most participants had lately recognized their pregnancy, they had all visited public health facilities (hospitals and health centers) at different times for various reasons, including to confirm the pregnancy, for regular ANC follow-up, and to seek care for some pregnancy-related illness. However, only one participant reported having a regular ANC visit. Other than being asked for their marital/spousal profile, all participants revealed that they received no counseling from healthcare providers during their ANC visits, and there was no further discussion to trace their psychosocial problems or link them with any possible social support service.

## Discussion

Breaking up during pregnancy is uncommon, but it can't be totally ruled out; and when it happens, it puts a pregnant woman in a difficult situation that makes her vulnerable to physical and psychosocial health problems. In this regard, this study explored the psychosocial effects of partner relationship breakup during pregnancy in order to give insight into the issue by describing the lived experiences of pregnant women who experienced breakup. A pregnant woman in such circumstances faces extreme stress, financial strain, and discrimination. Concerns about future parenting roles are also common, and pregnant women struggle to cope with this multifaceted situation.

According to the findings of the study, pregnant women reported important indications and actions that implicate severe stress conditions. Even though other studies didn't assess the specific effect of relationship breakup during pregnancy, studies done in Ethiopia (32–34) reported a lack of partner support and unsupportive relationship are associated with antenatal anxiety and depression. Additionally, some other studies conducted in Ethiopia found that single marital status or the lack of a cohabiting partner is a significant predictor of suicide attempts among pregnant women (35, 36). When a pregnant woman encounters an unexpected breakup, she is separated not only from her partner but also from the father-to-be of her conceived child, making her condition difficult to cause distress. Besides the socioeconomic reasons, the pregnant woman's self-esteem may deteriorate in such circumstances due to her perceived negative social value by the community and fear of related discrimination. Perhaps this intense situation may stay longer, and if no support or counseling is provided, the pregnant woman may develop anxiety or depression, which can have serious health consequences during this critical period.

Furthermore, pregnant women have reported prejudice and discrimination related to their pregnancy and current spousal status. This finding is similar to the findings of another study done elsewhere (26). Prejudice and discrimination, according to the participant's report, are very common among pregnant women who were living as cohabiting couples before the breakup. This is because pregnancy outside of marriage is a cultural taboo in Ethiopia, as it is in most other societies, and such cohabiting relationships are usually not open to families and relatives. This is also a major source of distress for pregnant women. Another type of discrimination reported was being forced to quit a job and the difficulty of getting a new job while pregnant, which was also reported among Korean single mothers in another study (26). The severe stress condition may also be aggravated when a woman loses her job and finds it difficult to get another job, and some studies have found an association between employment status and antenatal depression (37, 38).

The other significant finding is the economic difficulties that pregnant women may face as a result of partner relationship breakup during pregnancy. Seven of the study participants were employed or working in low-income generating activities, and it was difficult for them to lead an independent life following the breakup. Financial problems usually operate together with challenges in finding job and maintaining an active working status due to the pregnancy condition. As a result of this complex interplay, a pregnant woman may seek social support, usually from family and close friends, or may be forced to enter shelters. Such economic adversities expose a pregnant woman to a high level stress, which can lead to severe anxiety or other mental disorders. This finding is also consistent with other quantitative study findings that identified low-income and financial problems as significant factors for antenatal depressive symptoms (37, 38). Women in Ethiopia, like most other low- and middle-income countries, lack economic empowerment, and it is common to be financially dependent on one's partner. And when a relationship terminates, especially during pregnancy, the chance of becoming vulnerable to the economic crisis is high, and the pregnancy impedes the pregnant woman's efforts to deal with such a challenge. Moreover, concerns about future parenting roles are common due to uncertainties in getting job, raising a child as a single parent, and the negative effects of the breakup on the child's future life.

To cope with the partner relationship breakup, some pregnant women sought social support from family, relatives, or close friends. Others preferred to aid organizations due to a lack of support from family or close friends, fear of possible prejudice and discrimination, and the intensely stressful situation. Many studies have also found that social support is important during pregnancy (1, 21–23, 37). Similarly, Chen et al. reported that the direct and indirect efects of intra-family support through positive coping styles were stronger in decreasing the risk of antenatal depression among Chinese pregnant women (39). When a pregnant woman faces a relationshio breakup, good support from family and close ones can be protective and contribute to active coping. However, as our study indicated, pregnant women in breakup situations may lack this social support due to cultural perceptions, forcing the women to seek help from other organizations.

Pregnant women in breakup cases revealed that healthcare facilities and providers' efforts to screen, counsel, and link these risk mothers to any social support service are very weak. Even if such pregnant women are at risk of developing anxiety and depression, the mental health component is often overlooked by HCPs in the delivery of ANC services, resulting in a failure to identify and recognize such high-risk women in need of therapeutic and supportive intervention. Other studies (1, 2) have found similar gaps in ANC service delivery. The importance of assessing mental and psychosocial health issues through integrating into existing antenatal care services and comprehensive maternal care approaches in clinical practice has been recognized (25, 40–42). Notably, as Health Centres and Hospitals are the primary points of contact for health care needs, ANC clinics and HCPs should consider special risk conditions for more comprehensive ANC and counsel pregnant women who encounter partner relationship breakup.

While the study tried to address a unique risk condition in pregnant women and is descriptive of the various psychosocial aspects, it is not without limitations. The short duration of the study and the small number of study participants limited the researchers from observing other contexts that would have provided a more detailed understanding of the issue, and some dimensions might have been missed. Another possible limitation could be language. Since the interviews were conducted in local language (Amharic), there is a risk of losing the original sense of meaning when translating the content into English.

## Conclusions

The findings of the study give insight into the multifaceted nature of the situation and how the psychological, social, and economic effects intertwine and affect a pregnant woman during such breakup circumstances in Ethiopia and other similar socioeconomic and cultural contexts. Thus, the study help to shed light on the issue for further examination of this unique situation during pregnancy in order to initiate interventions that will ultimately target such vulnerable pregnant women and, in general, to improve the health of pregnant women and their children. It also highlights the existing gaps in accommodating such special risk conditions in ANC service delivery, as well as the need for more comprehensive ANC service.

Based on the study findings, it is suggested that community-level Information, Education, and Communication be initiated by the health sector, responsible social sectors (such as Women and Children Affairs offices), and NGOs to aware the community about the psychosocial consequences of relationship breakup during pregnancy, address cultural norms and discrimination, and promote supportive environments for these vulnerable pregnant women. Women's empowerment activities and psychosocial support services should also be strengthened, and pregnant women should be educated and encouraged to seek counseling services in such difficult situations. It is also suggested that healthcare managers and maternal health program experts sensitize and effectively communicate with HCPs at the ANC clinic about such unique risk conditions in order to direct them to more comprehensive ANC that can address special ANC needs. As far as knowledge of the researchers is concerned, no other study has investigated the effects of partner relationship breakup during pregnancy, and more research is needed to assess the effects of partner relationship breakup on pregnancy outcomes and its short and long-term effects on maternal and child health.

## Data Availability

The original contributions presented in the study are included in the study and supplementary materials, further inquiries can be directed to the corresponding author.
