# Supplementary material for: A phenomenological study of the lived experiences of partner relationship breakup during pregnancy: Psychosocial effects, coping mechanisms, and the healthcare providers' role
Source: Front Glob Womens Health. 2023 Apr 17;4:1048366. doi: 10.3389/fgwh.2023.1048366 (PMC10150961; doi:10.3389/fgwh.2023.1048366)
Supplement: Supplementary file 1 [file Table1.docx]

**Supplementary material**

**Supplementary file 1:** Topic guide used to explore the lived experiences of partner relationship breakup during pregnancy

1. How were your shared life and relationship with your partner/spouse before the breakup? Probe: Was the pregnancy planned? How do you remember the time when you recognized that you were pregnant?
2. How would you describe the situation when you finally breakup with your partner? Probe: How was the condition? Was that the most difficult part of your pregnancy?
3. How do you feel about the relationship breakup? Probe: Have you experienced any changes in your emotional or behavioral condition?
4. What challenges are you experiencing with your pregnancy? Probe: How is your living condition?
5. With whom do you discuss and share your feelings? What do you discuss?
6. What are the most supportive things for your current life?
7. What do you think about your childbirth and future parenting roles?
8. Have you visited a health facility for Antenatal care service? Probe: Did the health care provider(s) give you any counselling or support in relation to your situation?
