# Supplementary material for: A phenomenological study of the lived experiences of partner relationship breakup during pregnancy: Psychosocial effects, coping mechanisms, and the healthcare providers' role
Source: Front Glob Womens Health. 2023 Apr 17;4:1048366. doi: 10.3389/fgwh.2023.1048366 (PMC10150961; doi:10.3389/fgwh.2023.1048366)
Supplement: Supplementary file 2 [file Table2.doc]

**Supplementary Table 2: Codebook for describ**ing the psycho-social effects of partner relationship breakup during pregnancy

| **Code name** | **Code description** |
| --- | --- |
| Thinking of abortion at a mature stage of the pregnancy | *Considerable attempts to abort or terminate the pregnancy, despite knowing the late stage of the pregnancy and the potential risks.* |
| Suicide attempt | *Suicide attempt(s) as a result of a relationship breakup during pregnancy and feeling desperate about one's situation.* |
| Fear of sharing feelings with one’s family and close friends | *Fear of disclosing one's pregnancy status due to negative expectations that people and close friends will think of one as unfaithful and will not understand the situation; not sharing real feelings and concerns due to uncaring situations.* |
| Feelings of shame, guilt, and self-disgrace | *Loss of confidence in freely interacting with others; pregnancy-related self-stigma; fears and beliefs that others will judge the pregnancy negatively.* |
| Feelings of disregard and loneliness | *A decline in self-esteem; feeling ignored; desire to spend more time alone* |
| Attempting to spend the pregnancy time secretly | *Hiding oneself from family or friends in order to keep one's pregnancy hidden from others; attempting to find a place to spend the pregnancy time away from family and close social environment* |
| Psychological and emotional distress | *Loss of interest in interacting with others (e.g., spending more time sleeping and worrying about one's condition); desperate feelings* |
| Prejudice and discrimination | *Negative perceptions and attitudes towards the pregnancy; uncaring and unsupportive behavior; blaming for the pregnancy condition* |
| Difficulty in getting suitable work | *Finding job becomes challenging as the pregnancy matures and become visible; unable to maintain active working status due to the pregnancy condition.* |
| Job discrimination | *Employers refuse to allow pregnant women to work; employers directly or indirectly force women to leave jobs when employers recognize a woman's pregnancy; lack of consideration for making working environments supportive.* |
| Financial burden | *Financial struggles for living; income and job insecurity to cover living expenses; the effects of the partner breakup on one's life* |
| Forced to be in shelters | *Exhausted of all options for living independently or getting support from family/friends; a chance of getting oneself in a desperate situation, such as being a vagrant.* |
| Concerns about future parenting responsibilities | *Concerns about raising a child as a single parent, including how to meet future child needs; concerns about the future psychological impact of raising a child as a single parent on the child's personality development.* |
| Concerns about future uncertainties | *Uncertain about finding a suitable job after childbirth to lead an independent life* |
| Coping action cues | *Convincing oneself of the situation; actions taken to manage the breakup situation and its consequences during pregnancy* |
| Failure of healthcare providers to trace the intense stress condition during Antenatal care visits | *Didn’t counseled by healthcare provider regarding their condition; failure to detect the situation; unresponsive to conduct further assessment, including mental health assessment, for any therapeutic or preventive interventions; failure to link the pregnant women with any possible psycho-social support service* |
